# Analysis of a structured intronic region of the LMP2 pre-mRNA from EBV reveals associations with human regulatory proteins and nuclear actin

**DOI:** 10.1186/s13104-019-4070-1

**Published:** 2019-01-18

**Authors:** Nuwanthika Kumarasinghe, Walter N. Moss

**Affiliations:** Roy J. Carver Department of Biochemistry, Biophysics, and Molecular Biology, Iowa State University, 2437 Pammel Drive, Ames, IA 50011 USA

**Keywords:** Epstein–Barr virus, EBV, LMP2, Herpes virus, RNA, Splicing, Actin, hnRNP U, hnRNP A1, HuR, PSF

## Abstract

**Objective:**

The pre-mRNA of the Epstein–Barr virus LMP2 (latent membrane protein 2) has a region of unusual RNA structure that partially spans two consecutive exons and the entire intervening intron; suggesting RNA folding might affect splicing—particularly via interactions with human regulatory proteins. To better understand the roles of protein associations with this structured intronic region, we undertook a combined bioinformatics (motif searching) and experimental analysis (biotin pulldowns and RNA immunoprecipitations) of protein binding.

**Result:**

Characterization of the ribonucleoprotein composition of this region revealed several human proteins as interactors: regulatory proteins hnRNP A1 (heterogeneous nuclear ribonucleoprotein A1), hnRNP U, HuR (human antigen R), and PSF (polypyrimidine tract-binding protein-associated splicing factor), as well as, unexpectedly, the cytoskeletal protein actin. Treatment of EBV-positive cells with drugs that alter actin polymerization specifically showed marked effects on splicing in this region. This suggests a potentially novel role for nuclear actin in regulation of viral RNA splicing.

**Electronic supplementary material:**

The online version of this article (10.1186/s13104-019-4070-1) contains supplementary material, which is available to authorized users.

## Introduction

EBV (Epstein–Barr virus) is a ubiquitous human herpes virus that infects ~ 95% of adults [1]. EBV is implicated in various cancers [2–4] and autoimmune diseases [5]. Host-virus interactions are crucial to infection and the emergence of disease [6]. The precise mechanisms for EBV-implicated pathogenesis remain unclear, making molecular studies of this virus an active area of research. One particularly important area of study are the roles of RNA *inter*molecular [7–10] and *intra*molecular structures [11, 12], which have been found to be important to EBV infection. Intronic regions of the LMP2 (latent membrane protein 2) gene in EBV were previously shown to be “hot spots” for stable and conserved RNA structure [12]; suggesting potential roles in splicing regulation in LMP2, which is essential in establishing latent infection [13]. In addition to the dominant isoforms of LMP2 (A and B), this gene possesses numerous, less abundant, minor isoforms generated from alternative splicing [14]; a process known to be influenced by intronic RNA structure [6]. The splice sites between LMP2 exons 7 and 8 are the only ones not utilized in alternative splicing and, interestingly, are the only splice sites to both be included within a single structural domain. Both are in helixes that form part of a 119 nt RNA structure that spans parts of both exons as well as the intervening intron.

The unusual stability and conservation of this fold [12] suggests its importance to EBV, as do the presence of two unusual UU/UU internal “loop” motifs that are adjacent to each splice site. These motifs are rare, however, a single UU internal loop was previously found within the ISS (intron splicing silencer) of HIV-1: an RNA structure important to splicing regulation [15]. The sequence/structure of this ISS motif was found to associate with the human regulatory protein hnRNP A1 (heterogeneous nuclear ribonucleoprotein A1). To understand how analogous interactions of human proteins with the LMP2 intronic structural region might be playing similar regulatory roles, we undertook a study to identify its RNP (ribonucleoprotein) composition.

## Main text

### Results and discussion

**Fig. 1:**
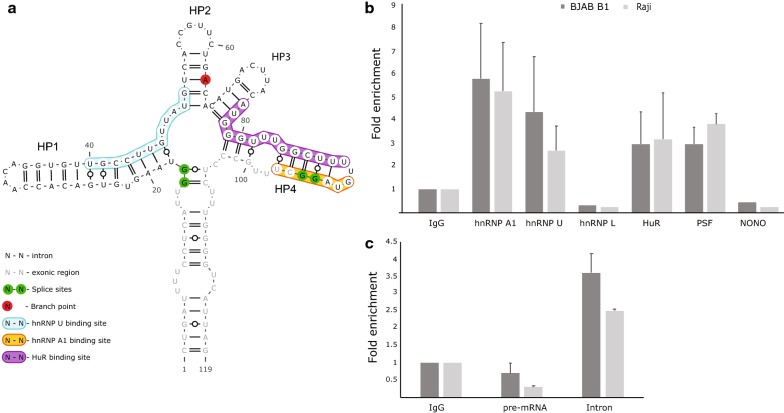
Validation of protein interactors. **a** Secondary structure model of the intronic sequence. hnRNP A1, hnRNP U and HuR binding sites predicted by RBPmap are color coded. **b** Fold enrichment of the LMP2 pre-mRNA following RIPs carried out with antibodies against hnRNP A1, hnRNP U, hnRNP L, HuR, PSF and NONO. **c** Fold enrichment of pre-mRNA (junction spanning primers) and intron (internal primers) following RIPs with anti-actin antibody. Data represents the average (with standard deviation) of independent experiments all normalized to control RIP with IgG. All RIPs (except for RIPs for NONO and hnRNP L) were carried out as either 2 or 3 independent experiments. All primer sequences used for the experiments are included in Additional file 6: Table S4

To identify putative protein interactors we utilized the program RBPmap to scan the 119 nt LMP2 intronic RNA structured region for human regulatory protein binding motifs [16]; complete results are in Additional file 1: Table S1. Three predicted interacting proteins (Fig. 1a) were validated using RNA immunoprecipitations (RIPs; performed in two EBV-positive cell lines, BJAB-B1 and Raji) followed by quantitative (q)PCR; the intronic region precipitated with HuR, hnRNP U and hnRNP A1 antibodies (Fig. 1b). HuR is involved in mRNA transport and affects post transcriptional modification via associations with additional regulatory proteins [17]. hnRNP U, also termed SAF-A (scaffold attachment factor A), interacts with a various pre-mRNAs, DNA elements with regions for nuclear matrix/scaffold attachment, and protein elements that include nuclear actin and the CTD (C-terminal domain) of RNA polymerase II. As mentioned previously, hnRNP A1 binds to a structured element within the HIV-1 ISS that, similar to EBV HP4 (Fig. 1a), forms a hairpin that contains a UU internal loop motif. In HP4, however, the UU/UU motif is closer to the hnRNP A1 binding motif and the 3′ splice site, which in HP4 overlaps this motif.

We also tested several other RNA-binding proteins that are predicted to be direct interactors or that might have protein–protein associations with HuR, hnRNP U or hnRNP A1. Although hnRNP L had a predicted binding site on the intronic structure, RIP data did not show any evidence of interaction (Fig. 1b). PSF (polypyrimidine tract-binding protein-associated splicing factor) and p54 nrb/NONO (Non-POU domain-containing octamer-binding protein), which may interact with hnRNP U, were also tested. While NONO failed to show any significant enrichment compared to the IgG control, PSF (involved in mRNA processing [18]) precipitated the intronic region (Fig. 1b).

To identify additional protein interactors that may not directly associate with the intronic region, a pull-down assay was performed using biotinylated “bait” RNAs to precipitate interacting proteins from human B cell lysates. Various sized proteins were pulled down under different wash stringencies (Additional file 2: Fig. S1); Mass spectrometry (MS) was used to identify two bands that appeared in both medium and high stringency conditions. Consistent with in silico predictions and RIP, one band (around 37 kDa; Additional file 1: Fig. S1) identified by MS was for hnRNP A1. Another band (37–50 kDa; Additional file 1: Fig. S1) was found to be very prominent, even under high stringency washes. When identified via MS, the highest confidence result was for actin (MS results are in Additional file 3: Table S2). RIP with anti-actin antibody confirmed that the intronic region was precipitated. Interestingly, only the intron between exon 7 and 8 could be amplified in the precipitated material (Fig. 1c). A fourfold enrichment (vs. IgG control) was observed when qPCR was carried out with primers amplifying the intron; however, unlike the RIPs for other interactors that precipitated both pre-mRNA (Fig. 1b) and intron (Additional file 4: Fig. S2), primers designed to amplify the intron–exon junction failed to show any significant enrichment in either BJAB-B1 or Raji cell lines. Actin should bind to both pre-mRNA and the intron sequence and the absence of pre-mRNA in actin pulldowns suggests that actin binding might promote splicing: e.g. the reaction occurs too quickly to capture the substrate with RIPs.

**Fig. 2:**
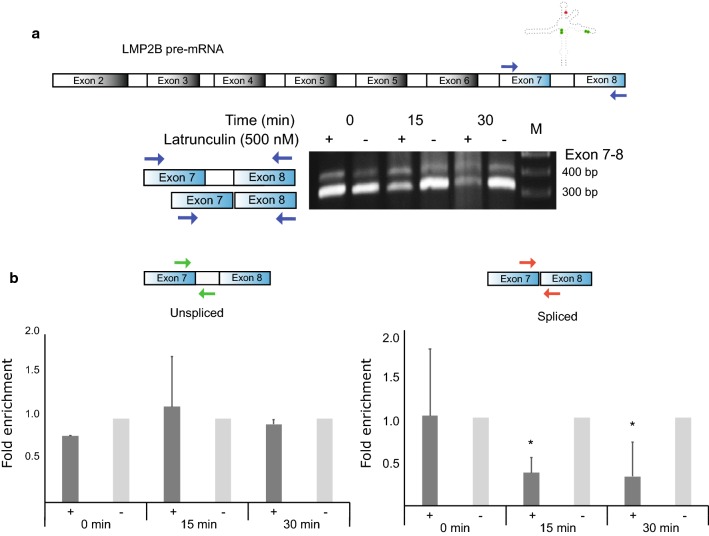
RT-PCR and qPCR analysis of spliced and unspliced transcripts following disruption of actin polymerization in BJAB B1 cells. **a** A cartoon of LMP2B with locations of exons RT-PCR primer sites and a model of the structured region is at the top. Below this are the results of RT-PCR analyses of spliced and unspliced transcripts in the presence of Latrunculin. **b** Spliced and unspliced variants quantified by qPCR analysis. Data shown are first normalized to housekeeping gene HPRT and plotted as a fold difference compared to the control at each time point. All data represents the mean (with standard deviation) from two independent experiments (*p < 0.05). All primer sequences used for the experiments are included in Additional file 6: Table S4

The association with actin was a surprise—nuclear actin is known to play roles in regulating transcription [19] and is hypothesized to affect mRNA maturation [20]; however, no roles for actin in the transcription or splicing of viral RNAs were previously reported. To determine if nuclear actin could affect splicing of the LMP2 intronic region, we assessed the effects of dysregulated actin polymerization on viral splicing. A drug known to interfere with actin polymerization in live cells was tested. Latrunculin sequesters free monomeric “globular” G-actin, inhibiting actin polymerization [21]. Consistent with a role for actin in stimulating splicing, the levels of unspliced transcripts remained relatively the same, while the spliced isoform levels showed a significant decrease over time via end-point RT-PCR (Fig. 2a) and RT-qPCR (Fig. 2b) in latrunculin treated BJAB-B1 cells.

This preliminary observation points to a potential role for nuclear actin in the regulation of EBV mRNA processing. Interactions with regulatory proteins may also be playing roles here. For example, a STRING analysis of validated interactors (Additional file 5: Table S3) finds an interaction network between hnRNP A1, hnRNP U and HuR, where HuR also has associations with actin. Additionally, hnRNP U was previously shown to interact with actin [18]. The roles of these protein–protein associations, as well as the interactions with the LMP2 intron sequence/structure (e.g. the UU/UU motif on HP4) require additional study. Future work will elucidate these roles, determine how wide-spread are the roles played by actin in EBV mRNA processing (and beyond), and help resolve to what extent actin’s role is co-transcriptional vs. post-transcriptional.

### Methods

#### Cell lines

Human Burkitt lymphoma B cell lines were generous gifts from Joan Steitz (BJAB-B1; Yale University)) and Siegfried Janz (Raji; University of Iowa). For cell culture work, RPMI growth medium supplemented with 2 mM l-glutamine, 1% penicillin–streptomycin, 10 mM Hepes, 1 mM sodium pyruvate (Life Technologies), and 10% FBS (Atlanta Biologicals) was used. Cells were maintained in a humidified incubator at 37 °C and 5% CO2.

#### Biotin pulldowns and mass spectrometry

The template for in vitro transcription of the full-length RNA was generated by PCR amplification. The oligonucleotides for HP4 template was purchased from IDT and the double stranded template was generated by heating to 95 °C then slow cooling to RT. The biotinylated RNA was synthesized using the T7 MAXIscript transcription kit (Invitrogen). After incubation, samples were DNase treated then purified by Phenol/Chloroform extraction.

Folding of the biotinylated RNA (3 µg) was done in a reaction volume of 45 µl (in water) by heating to 90 °C, then snap cooling on ice. After 5 min, the 10× RNA structure buffer (100 mM Tris pH 7, 1 M KCl, 100 mM MgCl_2_) was added and the RNA allowed to incubate at RT for 20 min. BJAB cells (~ 10^7^) were harvested and resuspended in 200 µl of RIP lysis buffer (50 mM Tris pH 7.4, 1 mM EDTA, 0.5% SDS). The cell suspension was sonicated briefly and 800 µl of RIP correction buffer (187.5 mM NaCl, 62.5 mM Tris pH 7.4, 1.75 mM EDTA, 1.25% NP-40, glycerol 12.5% and 1 mM PMSF) was added along with protease and RNase inhibitors (Thermo Scientific). The lysates were then nutated with the folded RNA for 1 h at 4 °C. Pre-cleared Pierce Streptavidin Agarose beads (50 µl) were added and nutated for another 1 h. Beads were spun down at 1000×*g* (2 min at 4 °C) and washed 3 times with 700 µl of cold NET buffer (150 mM NaCl, 50 mM Tris pH 7.4 and 0.05% NP-40).

Proteins were eluted from the beads by heating to 90 °C in 50 µl SDS gel loading buffer for 5 min and size fractionated on a 4–20% precast polyacrylamide gel (Bio Rad). Gels were silver stained using a silver stain kit (Pierce) and bands of interest excised. Excised bands were sent to the Protein Facility of the Iowa State University Office of Biotechnology for analysis via LC–MS/MS.

#### In vivo UV crosslinking and RNA immunoprecipitations

BJAB-B1 or Raji cells (~ 10^7^) were washed with ice cold PBS, resuspended in 2.5 ml PBS and irradiated in a 10-cm dish on ice with 254-nm UV light at 800 mJ/cm^2^. BJAB-B1 and Raji cell lysates were prepared using the same method described in the previous section. The lysates were pre-cleared by nutating with 20 μl Protein A/G-Sepharose (SantaCruz sc2003) for 1 h at 4 °C. For each immunoprecipitation, 3 µg of the following antibodies were used: p54nrb/NONO (sc376865), HNRNP U (sc32315), HuR (sc5261), HNRNPA1 (sc32301), PSF (sc101137), from Santa Cruz; β-Actin (622102) from Biolegend or HNRNPL (A303-895A) from Bethyl Laboratories. Control IPs were carried out with Normal Mouse IgG (sc2025) or normal rabbit IgG (Cell Signaling 2729 s). With the antibody, 20 µl of beads were added and incubated for 2 h at 4 °C. The beads were washed with NET buffer and I ml of Trizol (Invitrogen) was added to the beads. A 10% input was prepared by extraction RNA from 100 µl of lysate.

#### RNA extraction and qPCR

RNA was extracted according to the manufacturer’s instructions using Trizol. The RNA were DNase (M0303S; NEB) treated and concentrated using Zymo RNA Clean and Concentrator-5 (R1015) kit using its protocol. Reverse transcription (RT) reactions were performed with Superscript III using random hexamer primers (Invitrogen). For the RT reaction, the control RNA was diluted tenfold to prepare the 1% input. QPCR was performed using PowerUP Sybr mix and the QuanStudio3 instrument (Thermo Fisher). As the controls no-RT and no template reactions were included.

#### Actin assays

For the actin assays latrunculin (428020; Calbiochem) was used in concentrations reported previously (1). Following drug treatments, BJAB B1 or Raji cells (2 × 10^5^ cells) were isolated at three different time points and RNA was extracted using Trizol form the harvested cells. The gene expression levels were assessed by qPCR using the threshold cycle (ΔΔCT) method.

All primers used are listed in Additional file 6: Table S4.

#### Statistical analysis

The mean and standard deviation were calculated with Microsoft Excel software from independent experiments using biological replicates. The statistical significance was determined using the two-tailed Student’s t test.

## Limitations

The observations presented here are preliminary. Although we have validated several direct and indirect RNA–protein interactions in the intronic region, we are missing a map of the protein–protein interactions that form the RNP, we lack information on the exact roles of each interaction (in repression or stimulation of splicing) and the timing of these associations. We have suggestive results for actin, which point to a stimulatory roles for this association in splicing of this structured intronic region. However, we do not know how widespread are the effects of disrupting actin on splicing across EBV (and human) RNAs. These limitations, however, suggest many additional future analyses to parse out the roles of RNA structure and associations in LMP2 splicing and to better understand nuclear actin’s roles in regulating viral and host mRNA splicing.

## Additional files

**Additional file 1: Table S1.** RBPmap predictions of proteins predicted to bind motifs in the LMP2 structured intronic region.

**Additional file 2: Figure S1.** Silver stained gels from biotin pulldowns. Bands P553-02 and P636-01B were identified as actin and hnRNPA with LC/MS–MS. M—Marker, L—lysate, FL—Full length 119 bp RNA construct of the structured intronic region, HP4—truncated RNA construct containing only a 25 bp sub region HP4.

**Additional file 3: Table S2.** Mass spectrometry results on isolated silver stained bands (see Additional file 2: Fig. S1) from biotin pulldown experiments.

**Additional file 4: Figure S2.** qPCR data showing the fold enrichment of the LMP2 intron following RIPs carried out with antibodies against hnRNP A1, hnRNP U, hnRNP L, HuR, PSF and NONO. Data represents the average (with standard deviation) of independent experiments all normalized to control RIP with IgG. All RIPs (except for RIPs for NONO and hnRNP L) were carried out as either 2 or 3 independent experiments.

**Additional file 5: Table S3.** STRING analysis of validated protein interactors of the LMP2 structured intronic region.

**Additional file 6: Table S4.** List of primers.

## Abbreviations

CTD: C-terminal domain
EBV: Epstein–Barr virus
hnRNP A1: heterogeneous nuclear ribonucleoprotein A1
HuR: human antigen R
ISS: intron splicing silencer
LMP-2: latent membrane protein 2
MS: mass spectrometry
ncRNA: non-coding RNA
NONO: non-POU domain-containing octamer-binding protein
PSF: polypyrimidine tract-binding protein-associated splicing factor
qPCR: quantitative polymerase chain reaction
RBP: RNA binding protein
RIP: RNA immunoprecipitation
RNP: ribonucleoprotein
RT-PCR: reverse-transcriptase polymerase chain reaction
SAF: scaffold attachment factor A
